# Lyme Borreliosis: From Pathogenesis to Diagnosis and Treatment

**DOI:** 10.1155/2012/231657

**Published:** 2012-09-13

**Authors:** Joanna Zajkowska, Piotr Lewczuk, Franc Strle, Gerold Stanek

**Affiliations:** ^1^Medical University of Białystok, Białystok, Poland; ^2^Universitätsklinikum Erlangen, Erlangen, Germany; ^3^University Medical Centre Ljubljana, Ljubljana, Slovenia; ^4^Medical University of Vienna, Vienna, Austria

Since discovery of the etiologic agent of Lyme borreliosis (Lyme disease) in 1981 by Willy Burgdorfer, marked increases in knowledge of pathogenesis based on new technologies, more precise descriptions of common and seldom encountered clinical features, and significant progress in laboratory diagnosis have occurred. Ecological studies continue to contribute to the understanding of the disease epidemiology and new genospecies of *B. burgdorferi *sensu lato (s.l.) continue to be discovered. The geographical distribution of genospecies and their vectors and hosts explains some important differences in the clinical presentation of the disease. At least 5 genospecies of *B. burgdorferi *s.l. cause the disease in Europe while *B. burgdorferi *sensu stricto (s.s.) is the only *Borrelia* species pathogenic to humans in North America. In both continents, the same issues are still unresolved, such as the mechanisms for immunoevasion by the borreliae, the diagnostic challenges of current and historic infection, lack of effective vaccination, uneven diagnostic laboratory standardisation, and unsatisfactory therapeutic management. The persistence of symptoms in Lyme borreliosis patients following recommended antibiotic therapy continues to be controversial. New diagnostic approaches indicating ongoing infection are expected in patients with objective and subjective long-term sequelae of Lyme borreliosis. There are many current studies investigating new biomarkers or attempting to optimize existing ones. 

This special issue consists of articles highlighting recent advances in immunopathogenesis, diagnosis, and epidemiological approaches to Lyme borreliosis.

Lyme borreliosis is the most commonly reported tick-borne infection in Europe and North America, and despite the uneven reporting systems in different countries, it is apparent that Lyme borreliosis shows an increasing incidence. For good management, available clinical case definitions must be utilised. Speculation regarding links between *Borrelia *infection and a variety of nonspecific symptoms and disorders has resulted in over-diagnosis and over-treatment of suspected Lyme borreliosis, creating a challenge for health systems in many countries.

Arthritis is one of the main manifestations of Lyme borreliosis. A chronic, debilitating arthritis is classified as severe destructive Lyme arthritis. Antibiotic refractory Lyme arthritis (more often in the USA than in Europe) may result from *B. burgdorferi *s.l. induced autoimmunity in affected joints. This immune response might pose a potential problem in the production of a safe vaccine. Another problem is that there is no reliable medical laboratory marker for the diagnosis of Lyme arthritis. High levels of IgG antibodies to *B. burgdorferi *s.l. in serum are not sufficient for confirmation, especially in regions where there is high background seroprevalence. The excellent review article by *Erik Munson* and colleagues introduces experimental models of Lyme arthritis, clarifying the participation of mediators and molecular mechanisms of inflammation. Lessons from investigations on animal models of Lyme arthritis are required, but a universal animal model of Lyme borreliosis does not exist. It seems that the hamster model facilitates the assessment of severe destructive Lyme arthritis, while murine models allow investigation of immunopathology mechanisms.

To be protected from recognition and subsequent killing borreliae in the mammal host downregulate their surface proteins, hide in the extracellular matrix, and use complement neutralizing proteins such as Salp, CRASPs (complement regulator-acquiring surface proteins), or ISAC/IRAC, or induce the formation of immune complexes by secreting soluble antigens. The ability of the spirochete to neutralize complement allows it to survive and determines competent animal reservoirs associated with particular *B. burgdorferi* s.l. genospecies. CRASPs, presented on the surface of the spirochete differs in pathogenic genospecies of *B.burgdorferi *s.l. (CRASP-1, CRASP-2, CRASP-3, CRASP-4 and CRASP-5). *Claudia Hammerschmidt *and colleagues analyse the contribution of CRASP-4 in mediating resistance to complement in *B. burgdorferi *s.l. and the interaction with human complement regulators, indicating that CRASP-4 plays a subordinate role. 

*Iris Müller* and colleagues introduce a retrospective model of analysis evaluating frequency, diagnostic quality, and cost of Lyme borreliosis testing in Germany. The findings strongly suggest ongoing issues related to care for Lyme borreliosis and may help to improve future disease management. 

Why some individuals develop clinical manifestations after infection with *B. burgdorferi *s.l. while others remain asymptomatic is largely unknown. *Babro Skogman* and colleagues investigated adaptive and innate immune responsiveness to *B. burgdorferi *s.l. in Borrelia-antibody-positive asymptomatic children, children with previous clinical Lyme borreliosis, and unexposed controls. Lyme borreliosis in children follows a slightly different clinical course than in adults, and the duration of symptoms is often shorter. Children seem to have a better prognosis than adults and report persisting symptoms less often. The fact that some individuals may be infected with *B. burgdorferi *s.l. without developing clinical symptoms is interesting from an immunological standpoint and could indicate a more effective immune response to the spirochete in these individuals. In the conducted study, the adaptive and innate immune responsiveness to *B. burgdorferi *s.l. was similar in borreliae-infected asymptomatic children and children with previous clinical Lyme borreliosis. So, further studies on the immunological mechanisms of importance for eradicating the spirochete effectively are necessary. 

*John Lazarus* and colleagues consider in an excellent study whether current research methods, using either ELISA to detect seroconversion to *B. burgdorferi *s.s. antigens or PCR quantification of bacterial DNA within tissues, can accurately distinguish between an active infection and a past *B. burgdorferi *s.s. infection that was rapidly cleared by the innate responses. The results strongly indicate that both ELISA-based serological analyses and PCR-based methods can clearly distinguish between the two situations.

